# Silk fiber-based composite nanofiber tissue scaffold produced with electrospinning method

**DOI:** 10.3906/kim-2106-53

**Published:** 2021-10-05

**Authors:** Metin YÜKSEK, Mehmet AKALIN, Erhan SANCAK, Erkan İŞGÖREN, Onur ATAK, Derya SALTIK ÇİRKİN, Ali BEYİT

**Affiliations:** 1 Faculty of Technology, Textile Engineering, Marmara University, İstanbul, Turkey; 2 Institute of Pure and Applied Sciences, Faculty of Technology, Textile Engineering, Marmara University, İstanbul, Turkey

**Keywords:** Silk fibroin, polycaprolactone, polyethylene oxide, electrospinning

## Abstract

Wound dressings are one of the most rapidly expanding areas in medical textiles. In recent years, a large number of functional wound dressing has been developed. The aims of these products are to speed up the wound healing process and to provide maximum comfort for patients. Today, the multidisciplinary studies are required to improve further existed wound dressings. The results of such research studies are also promised new hopes for the Turkish textile industry weakened by the global competition.

For this purpose, three different polymers were selected as silk fibroin (SF) / polycaprolactone (PCL) / polyethylene oxide (PEO). The productions were made using triple and doublex mixture polymers and single polymer. It has been observed that the SF/PCL/PEO solution has extremely good viscosity, and conductivity values, fiber diameter, and structure thickness have the highest values. SF/PCL/PEO structure has a middle level in the strength value. It has been observed that the PCL polymer plays an effective role in the strength value. The most effective result for S group mats was seen in S2 (PCL/PEO) mat with a 2.62% reduction against the HaCaT cell line. A nanofiber tissue scaffold was produced by the electrospinning method, and a structured candidate for use was obtained by improving performance.

## 1. Introduction

The polymer fibers have a wide range of applications, which consist of functional textile and composite support material production [1]. Wet spinning, dry spinning, and soft spinning techniques apply mechanical forces. These are traditional fiber production methods [2, 3]. The fibers finer than microns find some specific uses in the medical, electronics, and industrial sectors [4–7].

The electrospinning method is a versatile technique that includes fluid dynamics, polymer chemistry, basic physics, electrical physics, mechanical and textile engineering. Nanoscale fibers are obtained one step in this method. The operation can be summarized as the production of nanofibers under electrostatic forces by applying a high voltage to an electrode (needle) connected to the tip of the syringe filled with the polymer solution or melt to be drawn [8–11].

Surfaces with nanofibers produced by the electrospinning method effectively contribute to cell renewal by increasing cell attachment, proliferation, and differentiation due to their high porosity, high surface area, and ability to simulate extracellular matrix (ECM) [12–14]. The intensive porous structure provides an ideal environment for the cells growing on the tissue scaffold to perform activities such as nutrient transfer and removal of cellular wastes [11, 15].

Silk fibers consist of two fibroin filaments that the sericins combine. It uses after removing sericin on silk fibroin (SF) [16]. Silk fibroin is a natural biopolymer generally composed of alanine, serine, glycine, and 18 different types of amino acids. Due to its superior properties such as biocompatible, biodegradable, and low immunogenic, silk fibroin is considered to be one of the most viable candidate biological materials [17]. Besides these properties, silk fibroin has a low inflammatory reaction, flexibility, good anti-thrombogenic, and excellent mechanical properties [18].

Polycaprolactone (PCL) is a viscoelastic and malleable, isomer-free, and affordable synthetic polymer with a wide range of products as scaffolding. Due to its low melting point (60 °C), it is soluble in many solvents such as chloroform, dichloromethane, benzene, acetone, and dimethylformamide [19]. It is a polymer with biocompatible, slow biodegradability, nontoxicity, and good mechanical properties [20]. Because it is a hydrophobic polymer, it degrades slowly. Therefore, it preserves its physical properties for a long time and can be used in long-term drug release, intra-body implants, wound dressing, and tissue engineering [21, 22]. The combination of polycaprolactone and silk fibroin allows both to improve mechanical properties and improve biocompatibility and to overcome disadvantages. Two-dimensional (2D) wound dressing, cartilage, neural, bone tissue engineering, and three-dimensional (3D) scaffolds can be created [23].

Polyethylene oxide (PEO) is a nonionic homopolymer of ethylene oxide. It is the most widely studied water-soluble polymer [24]. It can also be dissolved with polar solvents. Besides, while showing low toxicity, good stability, and lubricity, it provides a wide viscosity range by mixing with water and other solvents [25].

Ju et al. investigated the effect of electrospinning silk fibroin nanostructure on wound healing. They made production by electrospinning method by mixing silk fibroin and polyethylene oxide. In the experiment performed on the animal model, they saw an improvement after 21 days in the burn wound using silk fibroin. In the medical bandage, they observed that the wound was still open after 28 days. The silk fibroin nanostructure accelerated the re-covering of epithelial tissue and wound closure in the burn wound [26].

Yuan et al. produced a scaffold using SF/PCL. They observed that the fibrous cell elongation was 236.46 ± 82 μm, the dominant fiber was fibroin (91% aligned), and the cell alignment was 6.57 *°* ± 4.45 *°*. They evaluated it as providing moderate cell cultivation and accelerating early-stage cell movement in bone disorders [27].

Rubert et al. produced a structure with coaxial fibers in the electrospinning method by using PCL/PEO. The tests performed showed that the structure is better than for the regeneration of connective tissue, strengthening, fibroblast growth, and controlled release. Thus, they concluded that it would be promising for long-term treatment in vivo studies [28].

In this study, the mechanical properties of surfaces prepared with different mixing ratios of SF/PCL/PEO polymers and produced by the electrospinning method will be compared with cell tests. The aim is to examine the usability of the obtained structures in tissue engineering.

## 2. Experimental

### 2.1. Materials selection

Cocoons of *Bombyx mori* silkworm were kindly from Kozabirlik. Sodium carbonate (Na_2_CO_3_), Ethanol (CH_3_CH_2_OH), Formic acid (HCOOH, 98%), and Calcium chloride unhydrate (CaCl_2_) were purchased from Merck. Cellulose dialysis membranes (molecular weight cut off, MCWO: 12000 – 14000), polycaprolactone (MW: 80000) and polyethylene oxide (MW: ~900000) were purchased from Sigma-Aldrich.

### 2.2. Method

#### 2.2.1. Obtaining silk fibroin

The silk cocoons were cleaned and boiled in 0.02 M sodium carbonate for 1 h and separated from the series. The silk fibroins were dried and dissolved in the ternary solution CaCl_2_ / H_2_O / C_2_H_6_O (molar ratio 1: 8: 2). The completely dissolved solution was dialyzed for three days by changing distilled water. After the dialyzed solution was centrifuged, it was dried on a low-temperature heater. Drying silk fibroin particles were dried in the oven for one hour, and the processes were completed.

#### 2.2.2. Preparation of solutions

The first solution was prepared by using SF/PCL/PEO polymers. It was dissolved in formic acid at a ratio of 24: 10: 1 by weight and 17.5% w/v for 2–3 h in the stirrer.

The second solution was prepared by using PCL/PEO polymers. It was dissolved in formic acid at a ratio of 10: 1 by weight and 5.5% w/v, respectively, in the mixer for 2–3 h.

The third solution was prepared by using SF/PEO polymers. It was dissolved in formic acid at a ratio of 24: 1 by weight and 12.5% w/v, respectively, in the mixer for 2–3 h.

The fourth solution was prepared by using SF/PCL polymers. It was dissolved in formic acid at a ratio of 24:10 and 17% w/v, respectively, in the mixer for 2–3 h.

The fifth solution was prepared by using PEO polymer. Since a sufficient viscosity of 0.5% w/v could not be achieved in the PEO polymer, 2% w/v was changed. It was dissolved in formic acid to 2% w/v for 2 hours on the stirrer.

The sixth solution was prepared by using PCL polymer. It was dissolved in formic acid at 5% w/v for 2 h in the stirrer.

The seventh solution was prepared by using SF polymer. It was dissolved in formic acid at 12% w/v for 2 h in the stirrer.

The names of samples and descriptions of samples are shown in Table 1.

#### 2.2.3. Electrospinning process

A 10 mL plastic syringe was filled with fibroin solution for electrospinning. The electrospinning device (Inovenso, NE300 Nanospinner) was used to produce nanofibers. Cylindrical collector surface was covered with grease-proof paper. The process parameters are shown in Table 2.

### 2.3. Characterization of nanofiber structure

#### 2.3.1. The viscosity and conductivity of polymer solutions

The viscosity of the polymer solutions was determined by using a viscometer (Brookfield DV-E Viscometer, USA). The viscosity measurement was performed with S21 spindle at 100 rpm. The conductivity of the polymer solutions was measured by a conductivity meter (WTW Cond 3110, Germany). All experiments were carried out at room temperature.

#### 2.3.2. SEM analysis

The morphology of the electrospinning nanofibers was analyzed with scanning electron microscope (SEM) images (VEGA3 TESCAN, Tescan Analytics Tescan Orsay Holding, Fuveau, France). Samples of 10 × 10 mm were taken for measurement. The samples were covered with gold. Image J software was used to measure fiber diameters for images. SEM images of samples were taken at ×1000, ×2000, ×5000, and ×10000 magnifications.

#### 2.3.3. Mechanical test

All nanofiber structures were cut to 50 × 10 mm (length × width) for the mechanical test. Instron 4411 universal test (Instron, Norwood, MA, USA) device was used to examine the mechanical properties of nanofiber structures. The piston speed was set at 30 mm/min.

The thickness of nanofiber structures was measured with a Mitutoyo Digital Thickness Comparator (Mitutoyo, Kawasaki, Japan). Thickness measurements were made at 30 different points in both vertical and horizontal directions.

#### 2.3.4. Fouirer transform infrared spectroscopy (FTIR)

In the FTIR (fourier transform infrared spectra) examination of the silk fibroin mats were obtained on Perkin Elmer UATR Two, Waltham, MA, USA. Samples of 10 × 10 mm were taken for measurement.

#### 2.3.5. In vitro cell viability analysis

The viability testing of membranes was carried out by adopting the ISO10993-5: 2009 method to membranes, which describes the cytotoxicity test of medical devices [29]. In summary, in experiments, HaCaT human keratinocyte healthy cell line was used to mimic skin tissue. The cells were counted and planted in 1 mL of Dulbecco’ s Modified Eagle Medium (DMEM) into a 24-plate with 50,000 cells. The cells were incubated for 24 h in a 37 °C, 5% carbon dioxide oven. The sterilization of the membranes prepared as a square in 3× 3 cm dimensions was carried out under UV light for 45 min, and the samples were placed in the wells in 2 repetitions with the help of sterile tweezers. Membranes were allowed to incubate for 24 h or 24, 48, and 72 h. Membranes were removed when the time came, using sterile tweezers. After adding 100 μL of 5 mg/mL Cell Proliferation Kit I (MTT) solution to the wells, it was left in the oven for 4 h. After removing the supernatants, 1 mL of Dimethyl sulfoxide (DMSO) was added and stirred on the shaker for 1 h. Measurements were taken using a microplate reader and cytotoxic analysis was performed by comparing with the values of the control group.

## 3. Results and discussion

### 3.1. The viscosity and conductivity of polymer solutions

All experiments were carried out at room temperature. All viscosity and conductivity values of polymer solutions are shown in Table 3.

According to the data shown in Table 3, it was observed that the viscosity of the solution was provided by SF and PCL polymers in the combination of SF/PCL/PEO (S1), and the conductivity of the solution increased with the SF polymer. Different viscosity and conductivity values have emerged with combinations of polymers.

### 3.2. Morphology of nanofiber structures

The fiber diameters and surface views of nanofiber structures were evaluated by SEM images.

**Figure 1:**
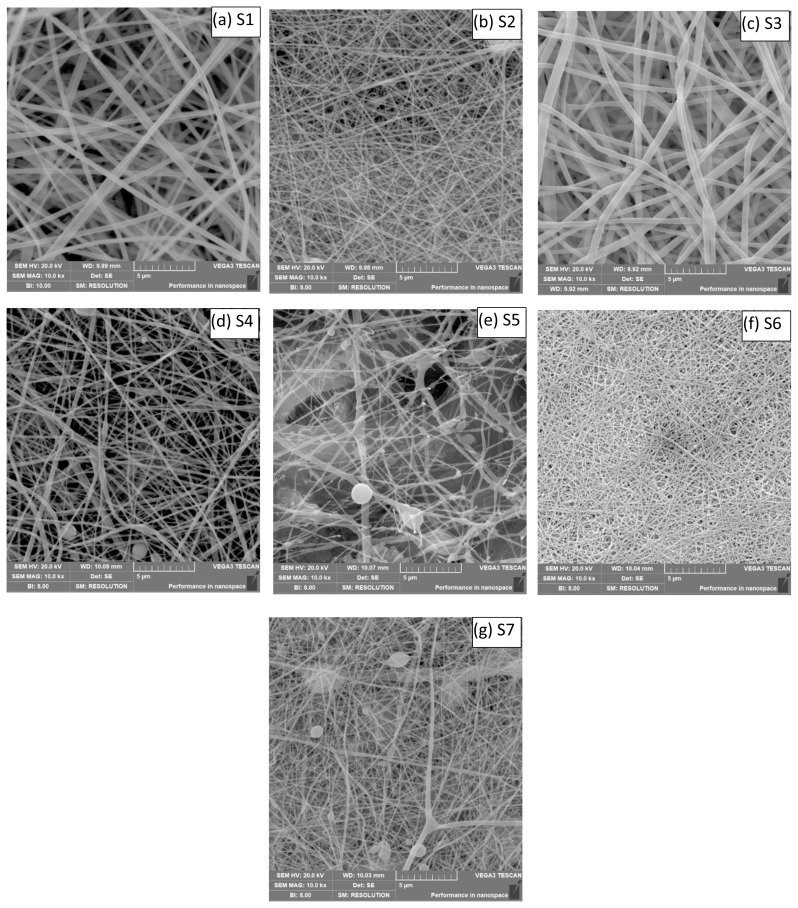
SEM images of nanofiber structures produced (magnification ratio 10.0 kx). ((a)S1, (b)S2, (c)S3, (d)S4, (e)S5, (f)S6 and (g)S7, respectively).

In the SEM images of the nanofiber structures shown in Figure 1, it was seen that there was fiber breakage in S5, a couple of pilling in S7, and fiber formation of other structures was continuous and uniform. Due to the low concentration of the PEO solution, breaking and pilling were observed in the nanofibers. It has been seen in a previous study that pilling formed by SF fibers can be produced continuously and uniformly by changing the electrospinning parameters [18].

The diameters of nanofiber structures were measured on SEM images by using the Image J programme. The diameters of nanofiber structures are shown in Table 4.

Among the nanofiber structures shown in Table 4, the S1 has the thickest fiber diameter. Depending on the mixtures of polymers, different thicknesses occurred in fiber diameters. These changes were observed in electrospinning parameters, due to the differences in the distance, the amount of feeding, and the applied tensions, in the fiber diameters of other structures. Electrospinning process parameters are also known to affect fiber diameters [30]. Therefore, the changed process parameters to achieve beadless fiber morphology may also have influenced the fiber diameter. Various studies supporting the results of this study have reported that the average fiber diameter increases with increasing the flow rate of the solution [31, 32]. In addition, the difference in fiber diameter may also be related to the change in viscosity and electrical conductivity of the electrospinning solution [33].

### 3.3. FTIR analysis

**Figure 2:**
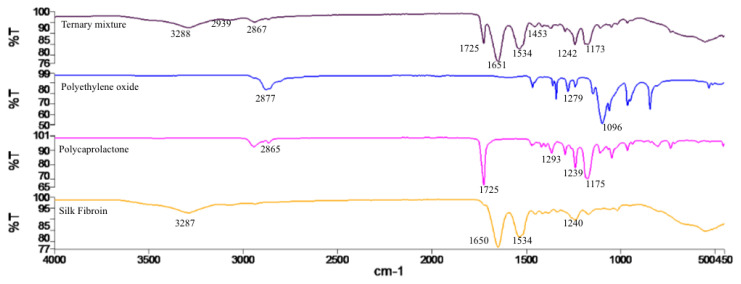
FTIR spectra of the ternary mixture, polyethylene oxide, polycaprolactone, and silk fibroin from produced structures.

FTIR spectra of the nanofiber structures produced are shown in Figure 2.

Due to the existence of amide groups in silk protein, the self-givenness pulse strips around 1620 cm^−1^ were allocated to the absorption peak of the peptide backbone of amide I (C=O stretching), the strips around 1230 and 1444 cm^−1^ to amide III (C-N stretching), strips around 1513 cm^−1^ to amide II (N-H bending) and 694 cm^−1^ to amide IV. Overall, these self-givenness absorbance peaks prognosticate the creature of a hydrogen-bonded NH group. The molecular structure of silk fibroin is characterized by β-sheet absorption peaks around 1630, 1530, and 1240 cm^−1^, superficial coil quality absorption peaks at 1650 or 1645, 1550, and 1230 cm^−1^, and an α-helix absorption peak around 1655 cm^−1^. The intensity of peaks around 3300 cm^−1^ undulates in reply to hydrogen bonds [34].

Polycaprolactone is found in FTIR spectrum as 2865 cm^−1^ symmetrical CH_2_ bond, 1727 cm^−1^ carbonyl bond, 1293 cm^−1^ crystalline phase C-O and C-C bond, 1240 cm^−1^ asymmetric COC bond, and 1170 cm^−1^ symmetrical COC bond [35].

Polyethyleneoxide is found in the FTIR spectrum as 2874, 1280 cm^−1^ C-H bond, and 1104 cm^−1^ C-O-C bond [36, 37].

According to FTIR results in Figure 2, polyethylene oxide was characterized by 2877 and 1279 cm^−1^ C-H bond, 1096 cm^−1^ C-O-C bond. Polycaprolactone was characterized by 2865 cm^−1^ symmetric CH_2_ bond, 1725 cm^−1^ carbonyl bond, 1293 cm^−1^ C-O and C-C bond in crystalline phase, 1239 cm^−1^ asymmetric COC bond, and 1175 cm^−1^ symmetric COC bond. Absorption peaks of 1650 cm^−1^ (amide I) and 1534 cm^−1^ (amide II), and 1240 cm^−1^ (amide III) forming silk fibroin irregular ring and α-helix structure were characterized. The characteristic peaks of the polyethylene oxide polymer were not observed in the FTIR spectrum of the structure produced due to a couple of polyethylene oxide in the membrane produced with the mixture using SF/PCL/PEO.[Table t5-turkjchem-45-6-1997]

### 3.4. Mechanical test

**Figure 3:**
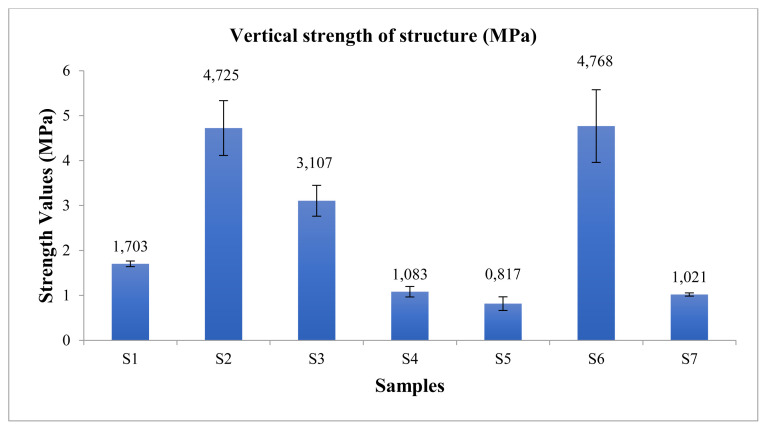
The vertical strength values of structure.

**Figure 4:**
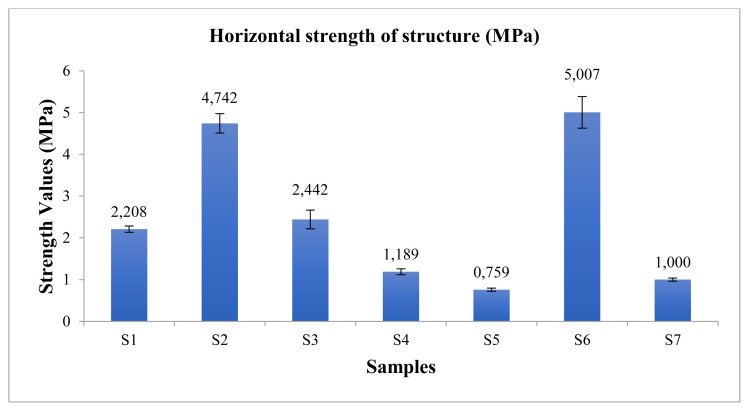
The horizontal strength values of structure.

The vertical and horizontal strength values of the produced nanofiber structures were measured (Figure 3 and Figure 4).

According to Figures 3 and 4, it was observed that there was a decrease in the strength of the structures where SF and PEO polymers were added. It has been observed that the PCL polymer is effective in strength in structures. Due to the viscoelastic and good mechanical properties of the PCL polymer [19, 20], it was observed that the S6 (PCL) mat had the highest strength value in the study. The strength of the obtained S1 is of medium strength by looking at all structures.

**Figure 5:**
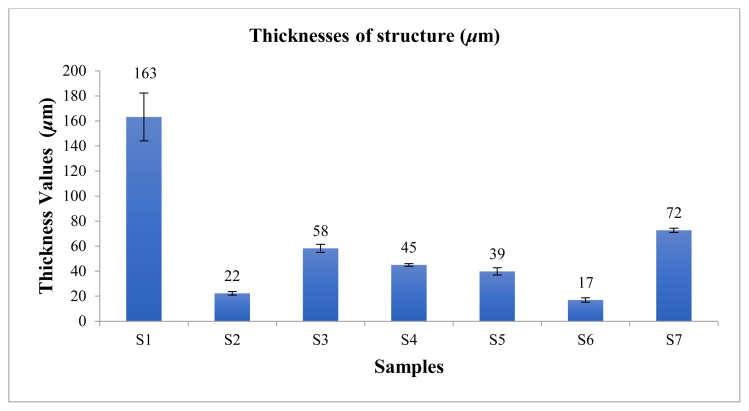
The thicknesses values of structures.

Structure thicknesses of the produced nanofiber structures were measured (Figure 5).

When the literature has been examined, a comparison with the thickness values of the nanofiber mats has not been found. The mat thickness is necessary information for the tensile strength test results. Since the tensile strength is shown as MPa in the literature, the thickness of the mat can be calculated here. It is provided as additional information in our research. Therefore, a comparison has been made to the obtained silk fibroin nanofiber mats. According to the values given in Figure 5, the structure created with SF/PCL/ PEO (S1) combination was the thickest structure. It was observed the difference in the structure thickness of other structures due to the combination of different polymers, solution viscosity, the distance from the electrospinning parameters, the amount of feeding, and the differences in the applied voltages.

### 3.5. In vitro cell viability analysis

**Figure 6:**
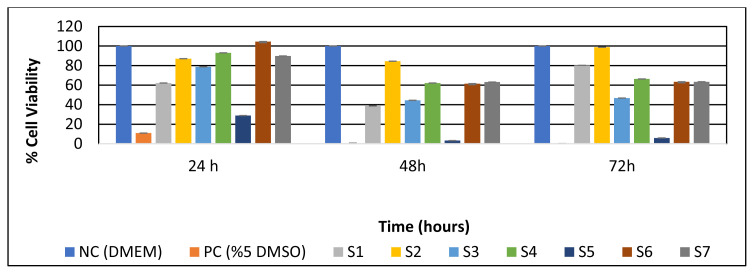
Determination of time-dependent cell viability of S group mats at 24, 48, and 72 h (DMEM was used in the negative control, 5% DMSO was used in the positive control).

In Figure 6, it was concluded that S1-S3-S4-S5-S6-S7 membranes preserve the viability of healthy human keratinocyte cells (HaCaT). Cytotoxic activity was observed at the end of the 48th hour for all S mats samples in time-dependent cytotoxicity experiments on S group mats. Cell viability was calculated as 38.61% for S1, 84.44% for S2, 44.42% for S3, 62.14% for S4, 3.19% for S5, 61.48% for S6, and 63.07% for S7. After 48 h, cells continued to proliferate until the end of 72 h. The most effective result for S group mats was seen in S2 (PCL/PEO) mat with a 2.62% reduction against the HaCaT cell line.

Kim et al. (2015), in their study with PCL and SF polymers, observed that no living cells were found in PCL microfibers, and that cell viability was higher in the composite formed with SF nanofibers [38]. In our study, although the PCL mat showed high viability in the first 24 h, PCL/SF mixed mats showed higher cell viability than the PCL mat at 48 and 72 h. Lin et al. (2016) performed biocompatibility tests by adding grape seed extract to silk fibroin and polyethylene oxide polymers. They observed 22.44% cell viability in the raw SF/PEO mats in the cell viability test. They observed that cell viability increased as the grape seed extract ratio increased [39]. In our study, cell viability in the SF/PEO mat was 46.72% at the end of 72 h.

## 4. Conclusion

In this study, silk fibroin (SF), polycaprolactone (PCL), and polyethylene oxide (PEO) polymers were used. The productions are made as triple and double mixtures and single polymer. Strength test, structure thickness, fiber diameter, structure surface imaging with scanning electron microscopy (SEM), and cell tests were performed on the produced structures.

As a result of the tests applied to the productions, it was observed that the viscosity of the solution was provided by SF and PCL polymers in the combination of SF/PCL/PEO (S1), and the conductivity of the solution increased with the SF. The first goal was to determine the optimum working conditions of the polymers with different properties used in the research. For this reason, it was necessary to make changes in the parameters depending on the electrospinning parameters, the properties of the polymers, the viscosity of the solutions prepared as single and mixture, conductivity, and the shaping of the Taylor cone formed during production. The highest voltage that can be applied in production in solutions with high solution conductivity and the lowest feed rate in solutions with low solution viscosity were determined. The collector distance was also modified to obtain a smooth Taylor cone and a continuous nanofiber structure without bits/bubbles. The reason for making changes in the parameters is to produce continuous fiber and a mat with high fiber smoothness by ensuring the continuity of nanofibers. When the SEM images of nanofiber structures were examined, it was seen that the formation of fibers was continuous, except for two structures. When the fiber diameters were examined, were measured the thickest fiber diameters in the S1. The difference in fiber diameters were measured, due to polymer mixtures in other structures, and different electrospinning parameters. The electrospinning process was made with SF/PCL/PEO mixture in S1 and PCL/PEO blend in S2. Since the blends in the S1 and S2 samples are different, it cannot wait that there will be a correlation between the S1 and S2 specimens. Since both materials have divergent properties (viscosity, conductivity), it was necessary to make changes in the electrospinning process parameters. Therefore, the obtained mats caused serious differences in fiber diameter, strength, structure thickness, and surface appearance properties. When the FTIR results are examined, SF, PCL, and PEO can be characterized individually. But the peaks of the polyethylene oxide polymer in the FTIR spectrum could not be observed, due to the small amount of polyethylene oxide used in the SF/PCL/PEO ternary mixture. When the strength values of the obtained structures were compared, it was observed that there was a decrease in the strength of the structures where SF and PEO polymers were added, and the PCL polymer was effective in the strength of the structures. When thicknesses of structures were measured, the thickest structure thickness was measured in the S1. The high variation in strength and structural thickness measurement values is because the prepared single, double and triple mixtures are different from each other. For this reason, it cannot be expected that there will be a correlation between all the samples obtained. Due to the different properties of the three polymers used, there were differences in fiber diameters, strength values, structure thicknesses, and surface appearance of the fibers.

After 48 h, cells continued to proliferate until the end of 72 hours. The most effective result for S group mats was seen in S2 (PCL/PEO) fabric with a 2.62% reduction against the HaCaT cell line. As a result of this study, the most suitable mat to be used in tissue applications was the S2 (PCL/PEO) structure. In particular, it was the most convenient structure because of the high cell viability at 72 hours, high strength, nanofiber size of the fiber diameter, and continuous fiber structure.

## Figures and Tables

**Table 1:** Names of samples and descriptions of samples.

Names of samples	Descriptions of samples
**S1**	SF/PCL/PEO
**S2**	PCL/PEO
**S3**	SF/PEO
**S4**	SF/PCL
**S5**	PEO
**S6**	PCL
**S7**	SF

**Table 2:** Electrospinning process parameters.

Samples	Applied voltage (kV)	Feeding rate (mL/h)	Distance	Velocity of collector (rpm)	Produce amount (mL)
**S1**	30	0,6	24	100	2.4
**S2**	32	0,8	20	100	2.4
**S3**	22	1,6	20	100	2.4
**S4**	30	0,6	24	100	2.4
**S5**	30	2	10	100	8
**S6**	35	0,6	20	100	2.4
**S7**	35	0,4	20	100	2.4

**Table 3:** The viscosity and conductivity values of polymer solutions.

Samples	Viscosity of solutions (cP)	Conductivity of solutions (*μ*S/cm)
**S1**	365	1220
**S2**	55	200
**S3**	151	1350
**S4**	214	1171
**S5**	414	275
**S6**	27	195
**S7**	76	1340

**Table 4:** The diameters of nanofiber structure (nm).

Samples	Diameters (nm)
**S1**	637 ± 302
**S2**	84 ± 17
**S3**	333 ± 100
**S4**	140 ± 51
**S5**	113 ± 36
**S6**	65 ± 17
**S7**	83 ± 26

**Table 5 t5-turkjchem-45-6-1997:** Determination of time-dependent cell viability of S group mats at 24, 48, and 72 h (DMEM was used in the negative control, 5% DMSO was used in the positive control).

	24 h	48h	72h
NC (DMEM)	%100,00	%100,00	%100,00
PC (%5 DMSO)	%10.84	%0.56	%0.11
S1 (SF/PCL/PEO)	%62.14	%38.61	%80.20
S2 (PCL/PEO)	%87.06	%84.44	%98.78
S3 (SF/PEO)	%78.64	%44.42	%46.72
S4 (SF/PCL)	%93.04	%62.14	%66.30
S5 (PEO)	%28.80	%3.19	%5.90
S6 (PCL)	%104.53	%61.48	%63.40
S7 (SF)	%89.81	%63.07	%63.40
